# From Cognitive Development to Intelligence: Translating Developmental Mental Milestones into Intellect

**DOI:** 10.3390/jintelligence5030030

**Published:** 2017-08-29

**Authors:** Andreas Demetriou, George Spanoudis

**Affiliations:** 1 Department of Psychology, University of Nicosia, Makedonitissas Avenue, P.O. Box 24005, 1700 Nicosia, Cyprus; 2 Department of Psychology, University of Cyprus, 1678 Nicosia, Cyprus; spanoud@ucy.ac.cy

**Keywords:** cognitive development, intelligence, individual differences

## Abstract

This special issue aimed to contribute to the unification of two disciplines focusing on cognition and intelligence: the psychology of cognitive development and the psychology of intelligence. The general principles of the organization and development of human intelligence are discussed first. Each paper is then summarized and discussed vis-à-vis these general principles. The implications for major theories of cognitive development and intelligence are briefly discussed.

## 1. Introduction

This special issue aimed to contribute to the unification of two disciplines focusing on cognition and intelligence: the psychology of cognitive development and the psychology of intelligence. Due to their epistemological origins and grounding, these disciplines developed in relative independence of each other. The founder of the first, Piaget, was a biologist with philosophical interests about the origins and nature of knowledge. It is well known that Piaget called his research program “genetic epistemology” to stress the developmental/evolutionary priorities of his research and the importance of using developmental research to answer philosophical questions about the nature and origins of knowledge. Luckily for us, at the beginning, the major source for research themes on cognitive development was the philosophy of knowledge: reasoning and logic, causality and scientific thought, mathematics and its laws, and concepts from the sciences, such as matter, space, and time. Regardless of how accurate Piaget’s original answers were, the field of cognitive development stayed in tune with many other disciplines throughout the years, such as cognitive and developmental science, focusing on the nature, emergence, and transformation of mental structures. As a result, one century of research on the development of thought in these domains generated a rich data base and several elaborate theories describing the state of understanding and problem solving in all realms as well as transition mechanisms driving change through the life-span. Piaget’s stage theory is probably the most well-known model of cognitive development. 

Psychology of intelligence developed in the tradition of understanding individual differences in various aspects of human behavior, including mental abilities. The main aim here was mapping mental abilities and their relations and measuring the standing of different individuals on them. Abilities in this tradition are considered as latent dimensions or traits underlying apparently disparate but actually related behaviours. Thus, the priority of research here was to uncover, delimit, and measure abilities rather than specify the mental processes involved. It is no coincidence that this tradition was very successful in delivering elaborate theories of the architecture of human intelligence and tests of intelligence that may be used to compare individuals in regard to mental abilities.

In current psychometric theory, the dominant model of the architecture of the human mind (see [1]) is a model integrating the Cattell and Horn [2] Gf–Gc theory with Carroll’s [3] three-stratum model. This common model is often referred to as the Cattell–Horn–Carroll model (CHC) model of intelligence. This model postulates that the human mind is organized in three hierarchical levels: a first level involves many situation-specific abilities or skills organized, at the second level, into eight broad abilities; each of these is identified by a key mental process shared by all first-level specific abilities (i.e., fluid intelligence, crystallized knowledge, general memory and learning, visual/spatial perception, auditory/verbal perception, retrieval ability, cognitive speediness, and processing speed). These in turn are constrained by general intelligence, or g, at the third level. Performance on the various tests of intelligence reflected by some widely known measures, such as IQ or the Raven test of fluid intelligence, are considered to closely represent g [2,3,4,5].

These two disciplines did not interact closely through the years despite their common focus on the human mind, despite calls to relate them as noted in the many of the papers in this special issue. Their weak interaction is reflected in the fact that so far there are no commonly accepted theoretical constructs that might unite the two disciplines. On the contrary: a promising construct that might bridge the two disciplines, mental age, was never integrated into cognitive developmental theories and it was eventually abandoned by psychometric theory. Mental age stands for an individual’s attainment on a test relative to the attainment of the “typical child” of each age (i.e., the majority of children in each year). When combined with the individual’s actual age, it yields the individual’s IQ, which was meant to be an index of its standing relative to his or her “typical age-mate” (i.e., “delayed”, “normal”, or “superior” (i.e., IQ = (MA/CA) × 100). Nowadays, MA was replaced by standard deviation, a purely statistical construct that may reflect age related accomplishments but it does not involve any developmental consideration. However, integration of the two fields may be very useful for both. Cognitive developmental research would acquire parsimony and accuracy in specifying constructs and their standing in the population. Psychometric research focusing on individual differences would be enriched by knowledge-based “categories of thought” and mechanisms of change, capturing aspects of mind so far left unnoticed and developing tests of ability that would be more sensitive to age-related priorities and characteristics. 

Despite limited direct interaction, in recent years, these two disciplines interacted through a third tradition studying the human mind: cognitive psychology. This tradition focused primarily on the more dynamic on-line aspects of mental functioning. The aim was to explain how humans (i) perceive the world and choose information that is relevant at a given moment; (ii) make sense of the information perceived; (iii) solve the problems encountered; and (iv) store and organize their knowledge and experience about the world. Information processing models dominated cognitive psychology since the early 1950s. In these models, controlled attention, processing speed, working memory, and inference are considered important in registering information, understanding, learning, and problem solving. 

Contributors to the special issue were asked to answer the following questions:

(1) Are there key constructs bridging cognitive development and intelligence, such as processing speed, executive control, working memory, reasoning, and reflection and awareness?
(2) How does the construct “cognitive development” relate to the construct “intelligence”? Are there distinct factors driving cognitive development and individual differences? Can we have a common metric of cognitive development and intelligence, such as an updated construct of mental age?
(3) How do general processes contribute to the formation of domain-specific ability and expertise and how does the acquisition of domain-specific ability and expertise contribute to the development and strengthening of general processes?

## 2. Key Constructs in the Mind

All papers presented in this special issue concur that there are common constructs that may be used to describe both cognitive development and individual differences in intellectual functioning. These may be described as a 4-fold architecture involving four types of mental processes: (1) domain-specific processes specializing in the representation and processing of specific types of information and relations in the environment, such as verbal, visuo-spatial, and numerical information; (2) representation processes, such as short-term storage capacity, allowing preservation and updating of information in service of further processing, or action; (3) reasoning processes, such as inductive and deductive reasoning, ensuring integration of information across time, senses, and choices, and evaluation for accuracy and validity; (4) cognizance ensuring awareness of choices and allowing evaluations according to past experience, the options available, and relevant criteria (logical, moral, interests, etc.). Highly popular processes, such as attention, executive control, and working memory are interactive products of the processes mentioned before: attention control, including inhibition, reflects awareness of a goal and a stimulus or an action; executive control requires awareness of a goal and a mental process, such as rehearsal in working memory that may be used to attain the goal.

## 3. Intelligence and Cognitive Development: Same or Different Dimensions?

For all authors of papers, intelligence is a phenotype interfacing the developing individual with the world. As such, it is primarily inference allowing meaning-making amidst incomplete or changing information and problem solving allowing goal attainment. Thus, it is primarily related to or fluid intelligence that grows with age and or experience. Tests, such as the WISC or Raven’s Matrices, primarily measure this phenotype. In psychometric terms, this is captured by psychometric g; in developmental terms, this is captured by a sequence of levels in thought development that an individual may ascend, à la Piaget or otherwise. Interestingly, there is widening consensus that there are distinct forces underlying cognitive development and individual differences in mental efficiency vis-à-vis real life goals. All papers in this special issue agree that mastering control is the major developmental force driving developmental ascension. Although emphasizing development from infancy to early adulthood, all papers bear implications for life-span intellectual development. Coyle [6] differentiates between decision (reaction) time (RT) and movement time (MT) in reaction time tasks and claims that the first reflects efficiency of information processing in the brain and the second reflects mastering control in executing a decision. Thus, the first stands for individual differences and the second stands for cognitive development. In his own words: “The RT–MT difference suggests that intelligence differences are attributable to cognitive processes in the brain, rather than motor processes in the periphery. Such a suggestion has developmental implications, since certain regions of the brain (e.g., the frontal lobes) are central for processing complex tasks, but are not fully developed until early adulthood, which suggests that the RT–MT difference may vary with development in childhood.”

The paper by Aeschlimann, Voelke, and Roebers [7] illustrates a more complex aspect of control: handling information in working memory (verbal digit and matrix visuo/spatial forward and backward) and how it relates to fluid intelligence (Cattel’s Culture Fair test comprising Series Completion, Classification, Matrix Completion, and Topological Reasoning). There are two notable findings: In the age span examined (9–11 years), the general working memory factor and the fluid intelligence factor are practical identical (0.95). When central executive processes underlying all working memory tasks were statistically isolated, the relation between the three of the four domain-specific working memory tasks and Gf was still significant, although moderate (in the 0.2–0.3 range). Therefore, working memory and inferential processes actually call upon the same central flexibility-updating-integration processes, using domain-specific representations as needed.

Anderson [8] expanded this argument further, arguing that general executive function is the major factor of cognitive development: “Developmental change is the province of mechanisms commonly regarded as components of executive functioning or cognitive control.” In full agreement with Coyle, Anderson postulates that individual differences are constrained by the speed of information processing. He minimally defines executive functioning as the ability for instantiating cognitive routines in dealing with hierarchies of task goals, which require monitoring ongoing information processing in sake of infallibly shifting between routines. He further suggested that the two factors are grounded in different aspects of the brain: Speed reflects white matter integrity, and it is primarily expressed via alpha rhythms in the brain and IQ reflecting individual differences. Executive function reflects grey matter connectivity, and it is primarily expressed via theta rhythms integrating alpha rhythms into hierarchies of activation. Thus, the concept of MA was justifiably abandoned because Binet was in error: Simply, individual differences and cognitive development cannot be placed along the same dimension.

This argument was explored further in our paper [9]. Combining two new methods for specifying relations between g and specific abilities, differentiation analysis and segmented modeling, we showed that the very nature of g changes with development. Although executive control is always present as a major pillar of mental functioning, it changes in both the type of representations involved and the type of reasoning that integrates them. Specifically, control of attentional focus is the major developmental priority in the cycle reality-based representations attained from 2 to 6 years of age. Awareness of the perceptual origins of knowledge emerges in the second part of this cycle, from 4 to 6 years. In the next cycle of rule-based thought, from 6 to 11 years, inductive reasoning dominates as the major marker of g. Awareness still actively infuses g with its properties, but it mutates from perceptual to the inferential aspects of representations, mainly in the second part from 9 to 11 years. In the next cycle of principle-based thought, from 11 to 17 years, inductive reasoning recedes and deductive reasoning, in its most advanced versions of conditional reasoning, dominates as the major source of infusion of new properties into g. In this cycle, awareness continues to be part of g re-morphing; however, it now comes as a refined theory of mental processes tuned to one’s own personal strengths and weaknesses, dominating primarily from 14 to 17 years. Therefore, psychometric g is never the same in development. It is re-morphed in each developmental cycle, because it is infused by different processes. The processes infusing g tend to intertwine with it and the processes that are consolidated tend to become freer from its influence.

It is noticeable that Ribaupierre and Lecerf [10] espouse a similar approach. They recognize basically the same fundamental mechanisms involved in g (processing capacity reflected in speed, inhibition reflected in attention control, and executive schemes weaved into various reasoning or problem solving chains). However, they argue that these processes are organized in a cascade fashion such that processing speed and inhibition account for working memory and working memory accounts for higher processes. Additionally, they present evidence that these mechanisms, although present at all ages, and in all individuals, vary in terms of their relative weight, depending on developmental phase, condition, and learning specific to a task. We have recently produced the evidence showing that, with age, in cascade models, the role of attention control and flexibility diminishes and reasoning and awareness increases [11,12]. Thus, the relative weight of these processes in different individuals may cause large inter- and intra-individual variability depending on the factors involved.

The interactive approach is best represented in this special issue by the paper contributed by van der Maas, Kan, Marsman, and Stevenson [13]. This paper builds on new methods of network modeling to extend van der Maas earlier conception of g as an interactive vector standing for the relations between the various mental processes rather than for any single process, such as attention, working memory, or fluid reasoning [14]. In the present paper, they extend this model defining g as a function of four factors: the mutual interactions between processes, the multiplier effects from the environment that can channelize and strengthen these interactions, the privileged role of some processes in the interactive network, such as working memory, that, if strong, may enhance the general interactive output, and the sampling in manifest test scores, which, if wide enough, as in the Raven test, may capture much of the interactive dynamics between processes.

In line with van der Maas et al. [13], our research showed that no specific process, such as inductive, deductive, spatial, or quantitative reasoning is a proxy of g. Nor can it be reduced to any fundamental process such as attention control, flexibility or shifting, or working memory. Interaction may be the way out of the impasse, but we suggest that it is not blind. It is directed by a mental mechanism holding the processes together and in liaison with the environment. We suggested that AACog (Abstraction, Alignment, and Cognizance) stands in the center of the 4-fold model, allowing and constraining the interaction of the four types of systems. Minimally, AACog allows search, inter-linking, and reduction of information or representations into new representations. AACog is the hard core of the language of thought (and natural language) because it allows its basic syntactic principles (compositionality, recursion, generativity, and hierarchical integration) to apply. AACog puts informational or representational patterns together (compositionality); it can take the patterns over and over again and embed them into each other (recursion); it can introduce variations in any of the patterns (generativity); it can embed compositions into reductions accounting for them (hierarchical integration). 

## 4. From Central and General to Localized and Specialized Intelligence

The AACog core mechanism can be translated into all sorts of intellectual ensembles, if domesticated in special domains by adding domain-specific qualifiers. We choose deductive reasoning as an example showing that a type of inference that appears general emerges as a specialization of translating the central core above into a representational ensemble of a specific set of relations in the environment that may also be expressed into a suitable syntax in language. For instance, in the cycle of episodic thought, we may see proto-deductive reasoning schemes in many infant behaviors, such as iteratively putting things together (conjunction) or choosing between things (disjunction). These formulate episodic blocks that will be mentally represented in the next cycle, yielding the pragmatic reasoning of the preschooler (you promised I could do this if I ate my food; I ate my food; so I can do this). When underlying rules are discovered, we get into a sequence ending in the ability to resist logical fallacies: it first appears as explicit conjunctive reasoning, handling “p and q” instances at 6–7 years (e.g., if my uncle’s car is in front of the house, my uncle is home; I see my uncle’s car; he is home); it then becomes biconditional reasoning handling “p and q” and “not p and not q” instances (e.g., my uncle’s car is not in front of the house; my uncle is not home); eventually, it evolves into full conditional reasoning handling “p and q,” “not p and not q,” and “p and not q” instances, and thus grasping the fallacies (e.g., my uncle may have left his car somewhere else; so I cannot conclude that he is not home just by not seeing his car in front of the house). 

It seems that, in development, a spatial contingency of “p and q” is mapped onto a time sequence recognizing that “p comes before q”, which is inferentially locked into a causal relation such that “p causes q”. Once consolidated as an inferential pattern, it is first inverted in that it is recognized that “when p is true then q is also true” implies that “when q is NOT true then p is also NOT true”; subsequently, it is recognized that these two forms are not equivalent in concern to their implications about each other: i.e., denying p is not informative about q and affirming q is not informative about p simply because q may be caused by other factors as well. In other words, p is embedded in an infinite set of other factors r, s, ..., i that may also operate causally like p in concern to q. This opens the mind to search for causal relations ad infinitum. 

Two complementary conclusions are suggested by this analysis. On the one hand, the system is fully functional at each developmental level. That is, all inferences possible at each of the levels above obey all four syntactic principles: they are compositional, recursive, generative, and hierarchical ad infinitum in that they can be applied to any instances of p and q. Additionally, some inferences, namely conjunctive inferences, are often correct, providing a functional frame for interpreting the world. On the other hand, each next level requires more executive power in two respects: Awareness of the total space of possibilities so that one holds decision until all possibilities are explicitly envisaged. This requires (i) an inhibition of the impulse to jump to conclusions; (ii) a strategy of efficiently filling in and revising/updating place holders until the entire inferential space is searched; and (iii) enough short-term storage space to represent all alternatives. 

In line with this analysis, Reverberi et al. [15] found that modus ponens is indeed unconscious in young college students. Specifically, they showed that students are primed to a modus conclusion even if a part of it is unconsciously activated. Notably, however, these researchers also found that disjunctive syllogism and the affirmation of the consequent fallacy are not automatic; no priming effect affected them, suggesting that both explicit awareness and processing of the relations are needed. College students are of course old enough to automate some pivotal components of conditional reasoning, such as modus ponens which operates as the background for building the rest of conditional reasoning, drawing upon a minimal amount of awareness, which allows the thinker to align alternatives with the internal mental truth-table blueprint mentioned above. Additionally, Barrouillet and Lecas [16] showed that the three levels above (conjunctive, biconditional, and conditional) are associated with mean working memory span of 3, 4, and 5 chunks, respectively.

This specialization may be seen in any domain. This is how mathematics is learned, how experimental thought is established, how meaning-making in reading is gradually constructed. That is, mental ensembles gradually drift away from g, as they become more complex and automated to be sufficient; automated to be efficient; and task-customized to be effective. The paper by Schneider and Niklas [17] is in line with this interpretation. They found longitudinally that IQ and working memory are reliable and strong predictors of academic achievement in various school subjects, such as reading, spelling, and mathematics. However, this relation varies with age. In early primary school years, the impact of these general factors on school subjects is strong; however, with growth earlier domain-specific attainments dominate in secondary school and college. 

## 5. Conclusions: Why Are Some People Smarter than Others?

In the introduction to this editorial, we noted that the authors were invited to specify the key constructs involved in intelligence, the constructs primarily driving cognitive development and those driving individual differences, and the interaction between general and domain-specific processes. Hopefully, the discussion above showed that there is considerable agreement between authors in answering these questions. They all agree that processing speed, executive control, working memory, reasoning, and reflection and awareness are important constituents of intelligence. The authors also agree that some processes, such as executive control and awareness, are more related to cognitive development; other processes, such as processing speed, are more related to individual differences in developmental rate and complexity of mental constructions. Finally, there is general agreement that general processes are more important to learning earlier in development. 

This very architecture explains why individuals differ in intelligence and learning possibilities. Specifically, individuals differ in all three important dimensions influencing intellectual functioning and development: genes, the brain, and the social environment. As noted in the paper by Rinaldi and Karmiloff-Smith [18], it is safe to conclude that there are several genes, which are explicitly related to several aspects of brain structure, functioning, and development; in turn, these aspects of brain functioning are explicitly related to several aspects of general mental ability, such as abstraction and handling novelty; in their turn, these are explicitly related to real world mental outcomes, such as IQ and educational attainment. There is an environment at all levels though: genes are expressed in a body, which functions in a nurturing (or under- or mal-nurturing) environment. Epigenesis then is an important force on its own. Brains interact with environments setting the experience and problem context in which they operate; mental processes take place in learning environments that contribute directly to their formation and development. Thus, differences in mind-related genes or the gene-related environment, brain structures, and brain-related environment, or the thought-related learning environment, do cause differences in intelligence and learning possibilities. 

How is this chain of effects translated into actual differences between individuals? The answer lies in the very nature of representations feeding into AACog at the successive cycles of development. Specifically, each successive cycle’s representations are more difficult to visualize by the mind’s eye. For instance, episodic representations have a compulsory nature of their own. Their properties are directly readable from the physical stimuli. Relations between them are part of their physical organization: the same color is physically present to the eyes; patterns in color or sound are physically present in their deployment in space or time. Thus, their alignment is directly guided by perceptual search; their abstraction only requires encoding the physical or pattern similarity; awareness of them may emerge by the time the individual reiterates what was seen or heard or turns to comparisons between matching episodes. 

Implementing AACog on realistic representations is already more difficult by several orders of magnitude. Abstraction over them requires holding them mentally together; this may go astray for several reasons, including external interference or forgetting; alignment requires mental search guided by an implicit rule used to hook relevant instances as they appear. Cognizance of representations requires their availability. Lack of any of them would cause delays in awareness and related metarepresentations that may be generated. For instance, lack of vision in congenitally blind 4-year-old children causes delays in the emergence of theory of mind [19]. 

Implementing rule-based AACog is more demanding in many respects. Abstraction here requires the relational shift that would direct search and alignment of relations across representations rather than properties in representations. The options for relating relations are by definition many more than relating representations because pairs of representations may be related in many alternative ways, depending upon their properties selected for processing (see also [10]). Awareness of inferential processes requires differentiating objects and representations from the mental processes applied on them. This is often difficult as indicated by the fact that children often focus on the content characteristics of tasks rather than underlying processes. 

Finally, implementing principle-based AACog is much more difficult than implementing rule-based AACog for the very reasons stated above. That is, abstracting over principles multiplies the options of abstracting over rules because principles bridge several rules. Awareness of inferential processes underlying principles requires by definition registering multiple processes. For instance, awareness of mental processes involved in conditional reasoning requires activating all four schemes discussed above. 

In conclusion, abstracting over representations and integrating them into higher levels of executive and reasoning schemes becomes increasingly difficult with increasing developmental level because options increase exponentially rendering errors more likely. In other words, decreasing likelihood of attaining later developmental levels is related to the very nature of the main factor of developmental transition in cognitive development. In line with this interpretation, the probability of attaining each successive level of intellectual development at the age modally associated with this level decreases in the general population. It is particularly notable that attainment of the cycle of principle-based thought is rare in the general population, limited to the upper 5% of the population at 11–12 years and the upper 25% at the age 16–17 years. Therefore, the sparsity of higher intelligence scores in the population is associated with developmental deceleration. That is, higher scores of intelligence require solving problems associated with later developmental phases. Therefore, high scores are constrained by developmental constraints. In the course to this ideal end-state speed may indeed reflect the ease and efficiency in affecting the translations needed in various domains, executive processes may reflect what is available for translation, and cognizance may indicate how good and refined the translation is going to be. 
